# Factors Associated With Declining Lung Cancer Screening After Discussion With a Physician in a Cohort of US Veterans

**DOI:** 10.1001/jamanetworkopen.2022.27126

**Published:** 2022-08-16

**Authors:** Eduardo R. Núñez, Tanner J. Caverly, Sanqian Zhang, Mark E. Glickman, Shirley X. Qian, Jacqueline H. Boudreau, Donald R. Miller, Christopher G. Slatore, Renda Soylemez Wiener

**Affiliations:** 1 Center for Healthcare Organization & Implementation Research, VA Boston Healthcare System, Boston, Massachusetts; 2 The Pulmonary Center, Boston University School of Medicine, Boston, Massachusetts; 3 VA Bedford Healthcare System, Bedford, Massachusetts; 4 VA Ann Arbor Healthcare System, Ann Arbor, Michigan; 5 University of Michigan Medical School, Ann Arbor; 6 National Center for Lung Cancer Screening, Veterans Health Administration, Washington, DC; 7 Department of Statistics, Harvard University, Cambridge, Massachusetts; 8 Center to Improve Veteran Involvement in Care, VA Portland Health Care System, Portland, Oregon; 9 Division of Pulmonary and Critical Care Medicine, Oregon Health & Science University, Portland

## Abstract

**Importance:**

Lung cancer screening (LCS) is underused in the US, particularly in underserved populations, and little is known about factors associated with declining LCS. Guidelines call for shared decision-making when LCS is offered to ensure informed, patient-centered decisions.

**Objective:**

To assess how frequently veterans decline LCS and examine factors associated with declining LCS.

**Design, Setting, and Participants:**

This retrospective cohort study included LCS-eligible US veterans who were offered LCS between January 1, 2013, and February 1, 2021, by a physician at 1 of 30 Veterans Health Administration (VHA) facilities that routinely used electronic health record clinical reminders documenting LCS eligibility and veterans’ decisions to accept or decline LCS. Data were obtained from the Veterans Affairs (VA) Corporate Data Warehouse or Medicare claims files from the VA Information Resource Center.

**Main Outcomes and Measures:**

The main outcome was documentation, in clinical reminders, that veterans declined LCS after a discussion with a physician. Logistic regression analyses with physicians and facilities as random effects were used to assess factors associated with declining LCS compared with agreeing to LCS.

**Results:**

Of 43 257 LCS-eligible veterans who were offered LCS (mean [SD] age, 64.7 [5.8] years), 95.9% were male, 84.2% were White, and 37.1% lived in a rural zip code; 32.0% declined screening. Veterans were less likely to decline LCS if they were younger (age 55-59 years: odds ratio [OR], 0.69; 95% CI, 0.64-0.74; age 60-64 years: OR, 0.80; 95% CI, 0.75-0.85), were Black (OR, 0.80; 95% CI, 0.73-0.87), were Hispanic (OR, 0.62; 95% CI, 0.49-0.78), did not have to make co-payments (OR, 0.92; 95% CI, 0.85-0.99), or had more frequent VHA health care utilization (outpatient: OR, 0.70; 95% CI, 0.67-0.72; emergency department: OR, 0.86; 95% CI, 0.80-0.92). Veterans were more likely to decline LCS if they were older (age 70-74 years: OR, 1.27; 95% CI, 1.19-1.37; age 75-80 years: OR, 1.93; 95% CI, 1.73-2.17), lived farther from a VHA screening facility (OR, 1.06; 95% CI, 1.03-1.08), had spent more days in long-term care (OR, 1.13; 95% CI, 1.07-1.19), had a higher Elixhauser Comorbidity Index score (OR, 1.04; 95% CI, 1.03-1.05), or had specific cardiovascular or mental health conditions (congestive heart failure: OR, 1.25; 95% CI, 1.12-1.39; stroke: OR, 1.14; 95% CI, 1.01-1.28; schizophrenia: OR, 1.87; 95% CI, 1.60-2.19). The physician and facility offering LCS accounted for 19% and 36% of the variation in declining LCS, respectively.

**Conclusions and Relevance:**

In this cohort study, older veterans with serious comorbidities were more likely to decline LCS and Black and Hispanic veterans were more likely to accept it. Variation in LCS decisions was accounted for more by the facility and physician offering LCS than by patient factors. These findings suggest that shared decision-making conversations in which patients play a central role in guiding care may enhance patient-centered care and address disparities in LCS.

## Key Points

**Question:**

What factors are associated with veterans declining lung cancer screening (LCS)?

**Findings:**

In this cohort study of 43 257 US veterans offered LCS, 32% declined; veterans who were older or had more severe comorbidity were more likely to decline screening, whereas Black and Hispanic veterans were more likely to accept it. The facility and physician offering LCS accounted for more variation in decisions than did patient factors.

**Meaning:**

The findings suggest that improving LCS discussions between patients and physicians could enhance patient-centered care and address disparities in LCS.

## Teaser

This cohort study investigates the frequency with which US veterans eligible for lung cancer screening accept or decline it and the patient- and facility-level factors associated with their decision.

## Introduction

Lung cancer is the leading cause of death from cancer worldwide, and lung cancer screening (LCS) has the potential to reduce mortality from lung cancer by approximately 20% among individuals at high risk.^1,2^ Lung cancer screening has been recommended by the US Preventive Services Task Force since 2013.^3,4^ However, fewer than 10% of eligible individuals have been screened, with lower rates of LCS in racial and ethnic minority populations.^5,6,7,8^

The decision of whether to screen individuals at high risk of lung cancer is complex and requires that patients and physicians balance the benefits (ie, early detection of lung cancer) with potential harms (eg, complications from downstream procedures, overdiagnosis, and distress from indeterminate findings).^9,10,11^ To address this concern, guidelines recommend that physicians engage in shared decision-making with patients about whether to undergo screening, and shared decision-making is mandated by the Centers for Medicare & Medicaid Services for reimbursement of LCS.^3,12^ Some researchers have suggested that these policies endorsing shared decision-making serve as a barrier to LCS uptake, arguing that policy requires too much discussion of LCS harms that may convince patients and/or the physicians counseling them that LCS is not worth pursuing.^13,14^ Of note, the 2022 Centers for Medicare & Medicaid Services policy continued to require shared decision-making when LCS is offered, reiterating the commitment to promoting patient-centered, informed decisions about LCS, and many physicians agreed that it was appropriate to continue the Centers for Medicare & Medicaid Services requirement for shared decision-making when LCS is offered.^15,16^

However, little is known about how frequently patients decline LCS when it is offered or factors associated with their decision. The Veterans Health Administration (VHA) provides an opportunity to explore these associations for a few reasons. First, the VHA was an early adopter of LCS and developed a standardized electronic health record (EHR) clinical reminder that prompts physicians to discuss LCS with eligible patients and indicate whether they accept or decline LCS. This clinical reminder is now used in multiple VHA facilities.^17^ Second, as the largest national health system in the US, the VHA provides cancer screening and care for millions of veterans from diverse geographic and sociodemographic backgrounds across multiple facilities, allowing exploration of the association of patient- and facility-level factors with LCS decisions. The objective of this study was to investigate how frequently patients declined LCS when it was offered by a physician and to assess patient- and facility-level factors associated with their decision. We hypothesized that there would be substantial variation in rates of accepting vs declining LCS across facilities and physicians. At the patient level, we hypothesized that older veterans with multiple cardiopulmonary comorbidities and that Black and Hispanic veterans would be more likely to decline LCS.

## Methods

### Study Design, Population, and Data Sources

**Figure 1.**
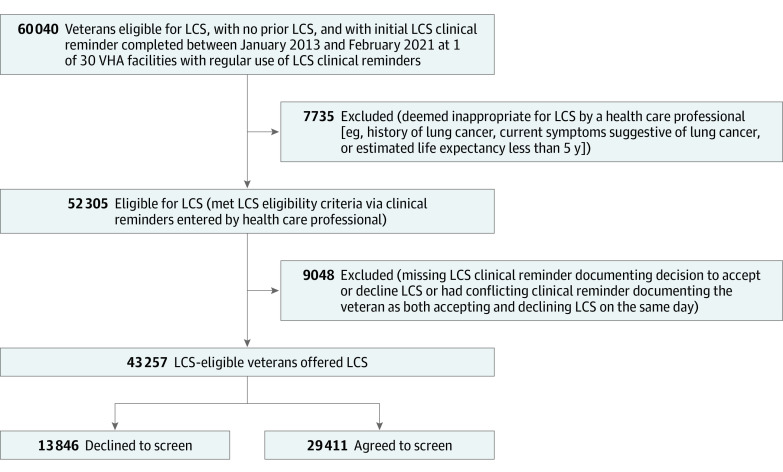
Study Cohort Derivation of US Veterans Eligible for Lung Cancer Screening (LCS) Who Either Accepted or Declined Screening VHA indicates Veterans Health Administration.

We conducted a retrospective cohort study of veterans who were (1) eligible for LCS based on 2013 US Preventive Services Task Force guidance (age 55-80 years, ≥30 pack-year smoking history, and either still smoking or quit ≤15 years ago) and (2) had documentation from clinical reminders that they were offered LCS and either accepted or declined it in any VHA facility from January 1, 2013, to February 1, 2021 (Figure 1). All study data were obtained from the Veterans Affairs (VA) Corporate Data Warehouse or Medicare claims files available through the VA Information Resource Center. Both LCS eligibility and the patient’s LCS decision were determined by presence of health factors generated by the EHR LCS clinical reminder. Given that a full shared decision-making discussion is recommended at the time of initiating LCS, we restricted our analysis to the first LCS decision documented in a clinical reminder for each patient. We excluded 193 veterans who had both “agree to LCS” and “decline LCS” documented on the same day; on the basis of medical record review of a random subset of 50 such veterans, many appeared to be accidental double entries. This study followed the Strengthening and Reporting of Observational Studies in Epidemiology (STROBE) reporting guideline for cohort studies and was approved by the VA Boston and VA Bedford Healthcare System institutional review boards, which deemed that informed consent was not applicable to the study.

### Study Setting

This study included VHA facilities that demonstrated regular use of the LCS clinical reminder in the EHR. We excluded facilities with low uptake of the LCS clinical reminder, defined as fewer than 300 LCS clinical reminders documented during the 6-year study period. The LCS clinical reminders are used to document LCS eligibility, whether LCS has been offered to eligible veterans, and a veteran’s decision to accept or decline screening. The LCS clinical reminder tool comprises a multistep process. First, veterans have their smoking history assessed during intake at a clinic visit, usually performed by a medical assistant or nurse. Second, if a veteran meets LCS eligibility based on age and smoking history, a clinical reminder will appear in the EHR prompting the physician to consider offering LCS. The physician then has the option of documenting the veteran as not appropriate for LCS (eg, symptoms of lung cancer, estimated life expectancy <5 years) or offering LCS in a shared decision-making conversation. The physician then documents whether the veteran agreed to or declined LCS.

### Primary Outcome and Covariates

The primary outcome was documentation (by clinical reminder) that a veteran declined LCS. We selected clinically relevant covariables a priori based on prior studies of factors related to uptake of or adherence to LCS, including self-reported race and ethnicity to explore potential disparities in declining LCS.^18,19,20,21,22,23^ For each veteran, we extracted data from the VA Corporate Data Warehouse on demographic characteristics and VA priority status (the VA’s system to determine co-payments). We used a veteran’s residential zip code to estimate median income, and we assessed urbanization using the US Census Bureau Rural-Urban Commuting Area codes.^24^ We defined comorbidities based on *International Classification of Diseases, Ninth Revision* and *International Statistical Classification of Diseases and Related Health Problems, Tenth Revision* codes in the VA Corporate Data Warehouse or Medicare files and calculated Elixhauser Comorbidity Index scores (eTable 1 in the Supplement).^25,26^ As a measure of health care utilization, the number of VHA outpatient and emergency department visits and the number of days in an inpatient hospital or skilled nursing facility in the year before the index date (the date of the clinical reminder documenting the decision to accept or decline LCS) were collected for each veteran. We performed log transformations of quantitative covariates that had right-skewed distributions including all the health care utilization covariables.

We assigned veterans to physicians based on the Corporate Data Warehouse physician identification number associated with the completed LCS clinical reminder. We assigned veterans to VHA facilities based on the facility where the LCS clinical reminder was documented.

### Statistical Analysis

We conducted hierarchical mixed-effects logistic regression analyses including both patient- and facility-level fixed effects to assess factors associated with declining LCS compared with agreeing to LCS. Our models assumed physician and facility random intercepts. We reported the mean patient effects, accounting for facility and physician random effects, in our 2-sided analyses. To quantify the variation in outcome attributable to facilities and physicians in our model, we calculated an intraclass correlation coefficient on the model with patient fixed effects and random intercepts for each facility and physician.^27^ Data were analyzed using SAS, version 9.4 (SAS Institute Inc).

#### Sensitivity Analysis

We performed a sensitivity analysis to explore the varying derivations of the study cohort. The primary cohort was derived from veterans who had a clinical reminder documenting them as eligible for LCS without any other clinical reminder documenting them as ineligible. However, there were some veterans who had conflicting eligibility—2 different clinical reminders documenting them as both eligible and ineligible for LCS (eg, if 2 health care team members entered smoking history differently). Therefore, we performed a sensitivity analysis including this population with conflicting eligibility in our multivariable model.

#### Medical Record Review

In another analysis, we performed medical record reviews of 60 randomly selected veterans who had a clinical reminder documenting them as either accepting (n = 30) or declining (n = 30) screening. We looked for documentation that supported their decision in the office visit notes that occurred on the date the decision was recorded.

## Results

Overall, there were 43 257 LCS-eligible veterans documented through clinical reminders as having been offered LCS; 29 411 (68.0%) agreed to screening, and 13 846 (32.0%) declined screening. Veterans in the sample had a mean (SD) age of 64.7 (5.8) years; 95.9% were male, 0.8% were American Indian or Alaska Native, 0.2% were Asian, 12.9% were Black, 0.3% were Hispanic, 0.5% were Native Hawaiian or Pacific Islander, 84.2% were White, 0.1% reported other race and ethnicity, 1.0% had unknown race and ethnicity, and 37.1% lived in rural zip codes. Veterans frequently had comorbid conditions, including 72.0% who reported currently smoking, 29.6% with chronic obstructive pulmonary disease (COPD), 24.5% with depression, and 23.6% with substance use disorder. At the facility level, a total of 30 facilities were included in the study, and 38.3% of veterans received their clinical reminder at a facility in the Southern US Census region. More than half of the veterans (58.6%) were offered LCS at a high-volume LCS facility, defined as performing more than 3000 screenings between 2015 and 2021 (Table). Veterans had similar distribution of comorbidities across facilities, without outliers. Of the 29 411 veterans who agreed to screening, 21 058 (71.6%) subsequently underwent LCS within the following 10 months compared with 483 of the 13 846 veterans (3.5%) who declined LCS, suggesting that the clinical reminders accurately captured veterans’ intentions regarding LCS.

**Table.** Characteristics of Veterans Who Accepted or Declined LCS After a Decision-Making Conversation With Their Practitioner

Characteristic	Veterans (N = 43 257)^a^	
	Accepted LCS (n = 29 411)	Declined LCS (n = 13 846)
**Individual level**		
Age, mean (SD), y	64.2 (5.7)	65.8 (5.9)
Sex		
Female	1268 (4.3)	493 (3.6)
Male	28 143 (95.7)	13 353 (96.4)
Race and ethnicity		
American Indian or Alaska Native	240 (0.82)	106 (0.77)
Asian	54 (0.18)	33 (0.24)
Black	4037 (13.7)	1536 (11.1)
Hispanic	92 (0.31)	22 (0.16)
Native Hawaiian or Pacific Islander	172 (0.58)	64 (0.46)
White	24 498 (83.3)	11 928 (86.2)
Other^b^	26 (0.09)	16 (0.12)
Unknown	292 (0.99)	141 (1.02)
Married	13 004 (44.2)	6619 (47.8)
Zip code–level income, median (IQR), $	48 757 (39 081-61 759)	49 214 (40 051-61 794)
Live in a rural zip code	10 505 (35.7)	5522 (39.9)
Distance to a VHA facility conducting clinical reminders, median (IQR), mi	25.6 (10.1-61.7)	31.0 (13.6-68.9)
VHA benefits (priority status)		
High disability with no co-payments	8881 (30.2)	3815 (27.6)
Low or moderate disability with partial co-payments	7124 (24.2)	3261 (23.6)
Limited disability with full co-payments	4483 (15.2)	2592 (18.7)
Poverty with no co-payments	8923 (30.3)	4177 (30.2)
Comorbid conditions		
Currently smoking	21 304 (72.4)	9855 (71.2)
History of major adverse cardiac event	3186 (10.8)	1475 (10.7)
Congestive heart failure	2067 (7.0)	1221 (8.8)
Stroke	1186 (4.0)	685 (4.9)
Chronic obstructive pulmonary disease	8652 (29.4)	4135 (29.9)
Interstitial lung disease	444 (1.5)	255 (1.8)
HIV infection	1128 (3.8)	384 (2.8)
Dementia	775 (2.6)	388 (2.8)
Depression	7749 (26.3)	2830 (20.4)
Posttraumatic stress disorder	4684 (15.9)	1827 (13.2)
Substance use disorder	7382 (25.1)	2822 (20.4)
Schizophrenia	740 (2.5)	397 (2.9)
Elixhauser Comorbidity Index score, mean (SD)	4.2 (3.2)	4.2 (3.3)
Health care utilization in the year before index date		
Outpatient visits, median (IQR), No.	11 (5-22)	7 (3-16)
Emergency department visits, median (IQR), No.	0 (0-1)	0 (0-0)
Inpatient length of stay, median (IQR), d	0 (0-0)	0 (0-0)
Long-term care facility length of stay, median (IQR), d	0 (0-0)	0 (0-0)
**Facility level**		
US Census region		
Northeast	7295 (24.8)	3580 (25.9)
Midwest	7195 (24.5)	2256 (16.3)
South	11 135 (37.9)	5421 (39.2)
West	3786 (12.9)	2589 (18.7)
LCS volume^c^		
Low	2129 (7.2)	1139 (8.2)
Medium	8984 (30.5)	5654 (40.7)
High	18 298 (62.2)	7053 (50.9)

Abbreviations: LCS, lung cancer screening; VHA, Veterans Health Administration.

^a^ Data are presented as the number (percentage) of participants unless otherwise indicated.

^b^ Other included individuals who reported “other” race or ethnicity.

^c^ Defined as the number of lung cancer screenings performed between 2015 and 2021. Low was defined as less than 1000 screenings, medium as 1000 to 3000 screenings, and high as more than 3000 screenings.

### Multivariable Model

In our multivariable model, veterans were less likely to decline screening if they were aged 55 to 59 years (odds ratio [OR], 0.69; 95% CI, 0.64-0.74) or 60 to 64 years (OR, 0.80; 95% CI, 0.75-0.85) compared with 65 to 69 years. In addition, veterans who were Black (OR, 0.80; 95% CI, 0.73-0.87) or Hispanic (OR, 0.62; 95% CI, 0.49-0.78) compared with White and veterans who reported currently smoking (OR, 0.90; 95% CI, 0.85-0.96) were also less likely to decline LCS. Veterans were more likely to decline LCS if they were older (age 70-74 years: OR, 1.27; 95% CI, 1.19-1.37; age 75-80 years: OR, 1.93; 95% CI, 1.73-2.17), had more comorbidities overall (OR, 1.04 [95% CI, 1.03-1.05] for every 1-point increase in Elixhauser Comorbidity Index score), or had specific cardiovascular or mental health comorbidities (history of stroke: OR, 1.14; 95% CI, 1.01-1.28; congestive heart failure: OR, 1.25; 95% CI, 1.12-1.39; schizophrenia: OR, 1.87; 95% CI, 1.60-2.19).

As a measure of VHA health care utilization, veterans with more frequent outpatient (OR, 0.70; 95% CI, 0.67-0.72) or emergency department (OR, 0.86; 95% CI, 0.80-0.92) visits were less likely to decline LCS, and veterans with more days spent at a long-term care facility were more likely to decline screening (OR, 1.13; 95% CI, 1.07-1.19). The associations among these log-transformed variables had a multiplicative interpretation. For example, every increase by a factor of the natural constant *e* (ie, 2.718) in the number of outpatient visits in the prior year corresponded to a 30% decrease in the odds of declining screening.

With regard to accessing care, compared with veterans who had to make co-payments to receive LCS, veterans with no co-payments owing to their income being below the poverty level (OR, 0.92; 95% CI, 0.85-0.99) or those with partial co-payments (OR, 0.89; 95% CI, 0.82-0.96) were less likely to decline screening. Meanwhile, veterans who lived farther away from the VHA screening facility were more likely to decline screening (OR, 1.06; 95% CI, 1.03-1.08).

**Figure 2.**
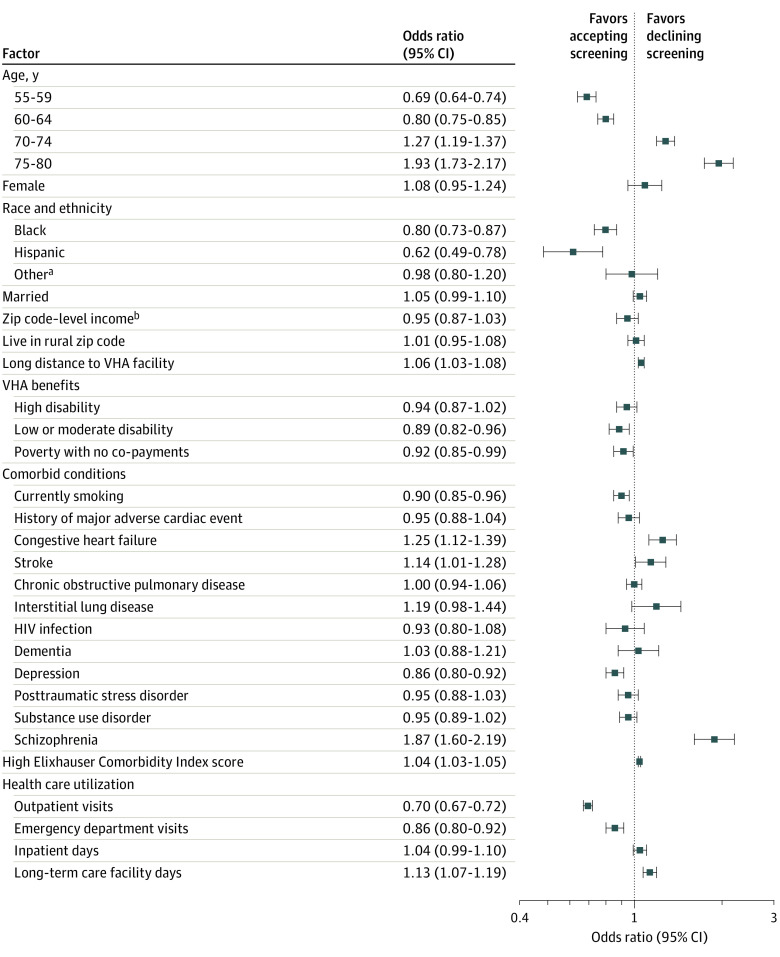
Patient-Level Factors Associated With Declining Lung Cancer Screening (LCS) in Multivariable Analysis Covariables included in the model that are not reported are body mass index, facility-level US Census region (East, South, Midwest, or West), and facility-level LCS volume (number of screenings between 2015 and 2021). Full model outputs are provided in eTable 2 in the Supplement. Squares represent odds ratios, with horizontal lines representing 95% CIs. VHA indicates Veterans Health Administration. ^a^Other race or ethnicity included American Indian or Alaska Native, Asian, Native Hawaiian or Pacific Islander, other, or unknown. ^b^Median income was estimated from the veteran’s residential zip code.

There were no observed differences associated with COPD, dementia, or living in a zip code with a low or high median income or a rural designation (Figure 2). In the multivariable model, there were no observed differences in the odds of declining LCS based on the geographic region of the facility or the number of LCS performed at that facility.

### Facility and Practitioner Variation

**Figure 3.**
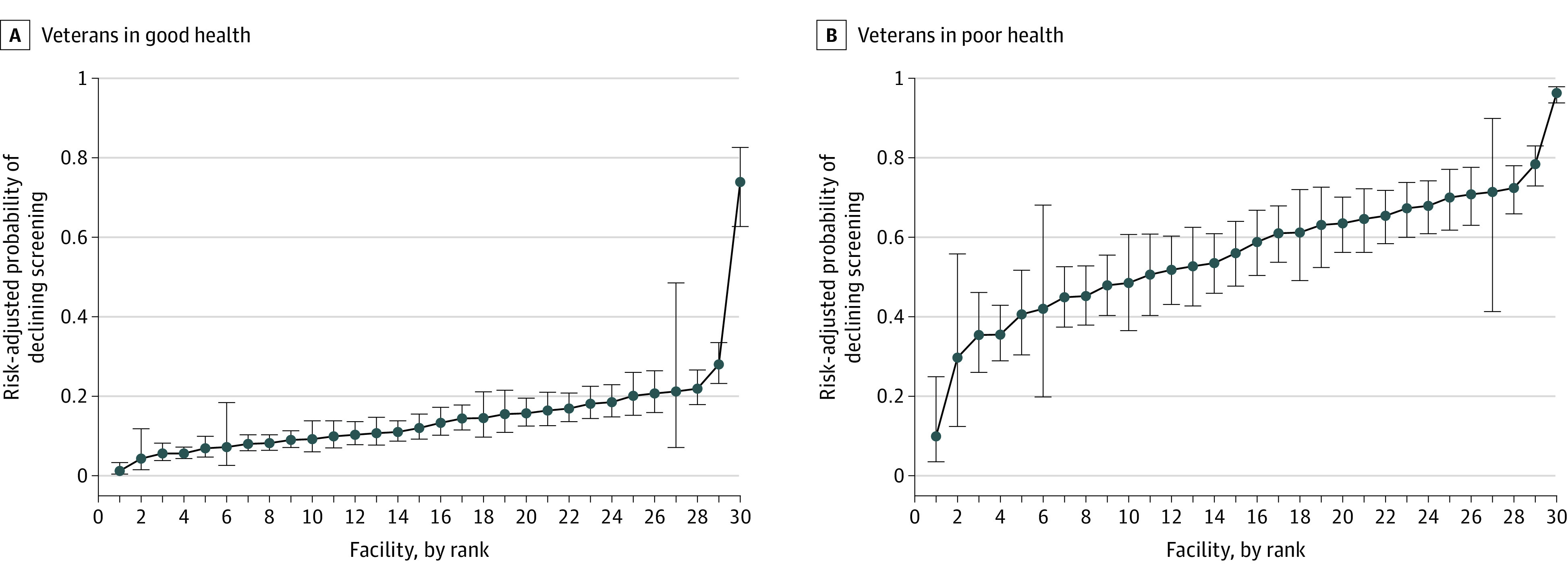
Facility-Level Variation in the Risk-Adjusted Probability of a Veteran Declining Lung Cancer Screening (LCS) Facilities (N = 30) are ranked by the risk-adjusted probability that patients would decline LCS. Whiskers represent 95% CIs.

We found that the probability of declining LCS in our cohort varied from 3.7% (95% CI, 0.8%-40.8%) to 61.8% (95% CI, 46.5%-75.1%) across VHA facilities (Figure 3). In our model with patient-level variables only, the facility at which a veteran received care accounted for 36% of the variation in the odds of declining LCS, and the physician who facilitated shared decision-making with the veteran accounted for 19% of the variation in the odds of declining LCS.

### Sensitivity Analysis

The sensitivity analysis including veterans who had conflicting documentation of eligibility showed an increase in the overall number of veterans in the cohort (N = 50 345). However, there were no changes in the direction of the point estimates of any of the exposure variables in the multivariable model (eTable 2 in the Supplement).

### Medical Record Review

Overall, 52 of the 60 veterans for whom a medical record review was conducted had no specific documentation of the conversation regarding LCS aside from the automatically generated template associated with the clinical reminder. Of the 8 veterans for whom clinic notes specifically mentioned LCS, 2 had agreed to screening and the clinic note confirmed their decision (eg, the patient agreed after shared decision-making using a decision aid tool). For the 6 veterans whose clinic notes confirmed that they declined screening, documented reasons included that the veteran was not interested in screening and that the veteran had more pressing issues; 1 veteran preferred to look into screening outside the VHA.

## Discussion

In this cohort study of US veterans eligible for LCS, 32.0% declined LCS after a discussion with a physician, with substantial variation across VHA facilities in rates of declining LCS. Of importance, the variation in declining LCS was accounted for more by the physician and facility at which the shared decision-making discussion occurred than by patient factors, a finding inconsistent with the goal of shared decision-making to increase the patient-centeredness of care. This finding suggests a need to improve the quality of patient-physician conversations about LCS by incorporating the core aspects of shared decision-making: (1) discussing the individualized benefits and harms of the medical options (to receive screening or not) with regard to the patient’s personal risk of lung cancer, (2) eliciting the patient’s values and preferences regarding these options, and (3) choosing the option that best matches the patient’s goals.^28^

We identified several patient characteristics associated with declining LCS. Veterans who were older or had more severe comorbidity, particularly those with serious mental health or cardiovascular comorbidities or those with skilled nursing facility stays, were more likely to decline LCS. This could reflect an appropriate consideration of potential harms outweighing potential benefits of LCS (ie, patients with multiple comorbidities may be less likely to benefit from LCS owing to limited life expectancy).^29,30,31^ Age is often a factor associated with decisions to pursue or forego cancer screening,^32^ but older age is also associated with a higher cancer risk and thus with a potentially higher chance of benefitting from screening. This complicates the role of age in decision-making about screening.

A key finding from our study was that groups that have experienced worse lung cancer care and outcomes, including Black and Hispanic individuals and those receiving full VA benefits owing to poverty, were more likely to accept screening, suggesting that screening may be a pathway to improving long-standing disparities in lung cancer.^33,34,35^ However, prior work has shown lower LCS rates among racial and ethnic minority groups and those with lower socioeconomic status,^7,8^ and we previously found lower adherence to recommended follow-up after initiating LCS among Black veterans and those with lower income.^18^ The current study adds to this literature by suggesting that disparities in LCS uptake and adherence may not be associated with screening hesitancy at the patient level but rather with structural barriers preventing receipt of equitable care. Our study found that overall, veterans with better access and connection to VHA care, as indicated by more VHA outpatient visits in the prior year, were more likely to accept LCS. In contrast, those who lived farther from a VHA facility or who had to provide co-payments were less likely to accept LCS. Future research is needed to identify and address barriers to LCS access and adherence to improve mortality overall and in underserved populations.

In addition to patient factors, we found that the physicians engaging in shared decision-making and the facilities at which veterans received their care accounted for a combined 55% of the variation in the probability of declining LCS. This large variation is greater than was previously reported in the VHA LCS demonstration project, the initial implementation of LCS across 8 VHA facilities, perhaps owing to the standardized approaches in the demonstration project, including LCS nurse coordinators and distribution of patient decision aids at all facilities.^17,23^ In contrast, the current study was conducted outside the period of the VHA demonstration project, encompassing a range of LCS practices and models (eg, centralized, hybrid, and decentralized) that arose organically across 30 facilities in clinical practice; such variation in the organization of LCS may influence shared decision-making implementation.^36^ The physician’s specialty and type are also associated with a patient’s decisions to pursue LCS,^37^ and physicians sometimes allow their own experiences, beliefs, and knowledge to influence the decision to screen for lung cancer rather than incorporating the patient’s preferences and values.^38^ In turn, patients reflecting on LCS discussions expressed trust in their physicians and often deferred to their physician’s recommendation whether or not to be screened, with some preferring a passive role in LCS decisions.^38,39,40^ However, multiple studies have shown that physicians tend to overemphasize the benefits of LCS with little mention of harms, do not use decision aids to facilitate patient understanding, and do not routinely explore patients’ values and preferences about LCS, all of which can hinder informed, values-based, and patient-centered decisions.^38,41,42,43^ Our study suggests that physician-dependent preferences and varying facility structures and processes (eg, centralized screening programs, LCS coordinators) may affect the decision to screen more than patient-specific factors. Future work is needed to determine which facility- and physician-level factors underlie this variation and to explore opportunities for improving patient-centered shared decision-making. For example, physicians may benefit from education reiterating that LCS discussions and subsequent decisions should take into account the patient’s personal risk of developing lung cancer, potential benefit from screening, and values and preferences.

### Strengths and Limitations

This study has strengths. It included 43 257 veterans cared for in 30 facilities, and to our knowledge, it is the largest study to examine factors associated with decisions to accept or decline LCS. This study was possible because of the VHA’s standardized clinical reminders to assess LCS decisions among veterans who were offered screening.

This study also has limitations. Some clinical reminders may have been completed inaccurately. For example, clinicians may have selected “patient declines LCS” as a shortcut to dismiss the reminder in the EHR when LCS was never offered owing to competing comorbidities or other reasons, as has been observed in other clinical contexts.^44^ To address this possibility, we performed a medical record review of clinical notes around the time of the decision for a random subset of veterans in this study. However, most notes did not document the details of the conversation, likely owing to physician time constraints. Nonetheless, that LCS was discussed and that the clinical reminder reflected the veteran’s intentions related to screening was supported by the subsequent receipt of LCS among those who accepted (71.6%) or declined (3.5%) screening. As a related limitation, our study was unable to assess the quality of the conversation during which LCS was offered (ie, whether shared decision-making occurred). However, the VHA recommends shared decision-making for LCS, and prior work has shown that VHA physicians discuss at least some of the elements of shared decision-making with patients.^38,45,46^ This study could not explore factors associated with patient decisions to decline LCS that were identified in prior qualitative and survey studies, such as lung cancer worry, perceptions of the benefits vs harms of LCS, fatalism, and logistic barriers to accessing LCS.^39,47,48,49^ Our findings may or may not be generalizable outside the VHA; for example, other studies showed that insurance coverage and cost of LCS and downstream care to patients were associated with patient willingness to accept LCS; these factors may be less relevant to veterans receiving care in the VHA.^48,50^

## Conclusions

Lung cancer screening is underused in the US both inside and outside the VHA, in part owing to a lack of awareness of LCS among populations at risk.^6^ The goal of shared decision-making conversations is to educate patients about LCS and its harms and benefits, consider patients’ individual lung cancer risk, and incorporate patient preferences and values with regard to LCS to improve overall LCS uptake and patient-centered care.^51^ In this cohort study, we found that the physician and site that offered LCS accounted for more variation in LCS decisions than did patient factors, suggesting a need to refocus shared decision-making conversations on the patient’s individual circumstances and standardize shared decision-making to minimize physician and facility variation.^13^ At the patient level, older veterans with serious comorbidities were more likely to decline LCS, suggesting that competing health demands for these patients may have made LCS a low priority. Black and Hispanic veterans and those receiving full VA benefits owing to poverty were more likely to accept LCS, suggesting that offering LCS to all those eligible and reducing or eliminating co-pays may help address long-standing disparities in lung cancer. Further action is needed to address barriers to LCS uptake and adherence in underserved populations to avoid exacerbating existing disparities.

## Notes

Supplement. **eTable 1.** *International Classification of Diseases, Ninth Revision (ICD-9)* and *International Statistical Classification of Diseases and Related Health Problems, Tenth Revision (ICD-10)* Current Procedural Terminology Codes for Comorbidities **eTable 2.** Multivariable Analysis of Primary Model Compared With Models Used in Sensitivity Analyses, Including Veterans With Conflicting Eligibility Documentation

## References

[1] Aberle DR, Adams AM, Berg CD, ; National Lung Screening Trial Research Team. Reduced lung-cancer mortality with low-dose computed tomographic screening. N Engl J Med. 2011;365(5):395-409. doi:10.1056/NEJMoa1102873 21714641 PMC4356534

[2] de Koning HJ, van der Aalst CM, de Jong PA, . Reduced lung-cancer mortality with volume CT screening in a randomized trial. N Engl J Med. 2020;382(6):503-513. doi:10.1056/NEJMoa1911793 31995683

[3] Krist AH, Davidson KW, Mangione CM, ; US Preventive Services Task Force. Screening for lung cancer: US Preventive Services Task Force recommendation statement. JAMA. 2021;325(10):962-970. doi:10.1001/jama.2021.1117 33687470

[4] Moyer VA; US Preventive Services Task Force. Screening for lung cancer: US Preventive Services Task Force recommendation statement. Ann Intern Med. 2014;160(5):330-338. doi:10.7326/M13-2771 24378917

[5] Lewis JA, Samuels LR, Denton J, . National lung cancer screening utilization trends in the Veterans Health Administration. J Natl Cancer Inst Cancer Spectr. 2020;4(5):pkaa053. doi:10.1093/jncics/pkaa053 33490864 PMC7583162

[6] Fedewa SA, Kazerooni EA, Studts JL, . State variation in low-dose computed tomography scanning for lung cancer screening in the United States. J Natl Cancer Inst. 2021;113(8):1044-1052. doi:10.1093/jnci/djaa170 33176362 PMC8328984

[7] Tailor TD, Tong BC, Gao J, Henderson LM, Choudhury KR, Rubin GD. Utilization of lung cancer screening in the Medicare fee-for-service population. Chest. 2020;158(5):2200-2210. doi:10.1016/j.chest.2020.05.592 32562612

[8] Sosa E, D’Souza G, Akhtar A, . Racial and socioeconomic disparities in lung cancer screening in the United States: a systematic review. CA Cancer J Clin. 2021;71(4):299-314. doi:10.3322/caac.21671 34015860 PMC8266751

[9] Zhao H, Xu Y, Huo J, Burks AC, Ost DE, Shih YT. Updated analysis of complication rates associated with invasive diagnostic procedures after lung cancer screening. JAMA Netw Open. 2020;3(12):e2029874. doi:10.1001/jamanetworkopen.2020.29874 33326023 PMC7745100

[10] Wiener RS, Gould MK, Woloshin S, Schwartz LM, Clark JA. “The thing is not knowing”: patients’ perspectives on surveillance of an indeterminate pulmonary nodule. Health Expect. 2015;18(3):355-365. doi:10.1111/hex.12036 23252477 PMC3880393

[11] Patz EF Jr, Pinsky P, Gatsonis C, ; NLST Overdiagnosis Manuscript Writing Team. Overdiagnosis in low-dose computed tomography screening for lung cancer. JAMA Intern Med. 2014;174(2):269-274. doi:10.1001/jamainternmed.2013.12738 24322569 PMC4040004

[12] Mazzone PJ, Silvestri GA, Souter LH, . Screening for lung cancer: CHEST guideline and expert panel report. Chest. 2021;160(5):e427-e494. doi:10.1016/j.chest.2021.06.063 34270968 PMC8727886

[13] Sands J, Tammemägi MC, Couraud S, . Lung screening benefits and challenges: a review of the data and outline for implementation. J Thorac Oncol. 2021;16(1):37-53. doi:10.1016/j.jtho.2020.10.127 33188913

[14] Grannis FW Jr, Ross S. Shared decision-making in lung cancer screening: whence? whither? *ASCO Post*. April 25, 2019. Accessed August 12, 2021. https://ascopost.com/issues/april-25-2019/shared-decision-making-in-lung-cancer-screening/

[15] Slatore CG, Golden SE, Thomas T, . Beliefs and practices of primary care providers regarding performing low-dose CT studies for lung cancer screening. Chest. 2022;161(3):853-859. doi:10.1016/j.chest.2021.08.062 34480957 PMC8941621

[16] Centers for Medicare & Medicaid Services. Screening for lung cancer with low dose computed tomography (LDCT): decision memo. February 5, 2015. Accessed July 24, 2021. https://www.cms.gov/medicare-coverage-database/view/ncacal-decision-memo.aspx?proposed=N&NCAId=274

[17] Kinsinger LS, Anderson C, Kim J, . Implementation of lung cancer screening in the Veterans Health Administration. JAMA Intern Med. 2017;177(3):399-406. doi:10.1001/jamainternmed.2016.9022 28135352

[18] Núñez ER, Caverly TJ, Zhang S, . Adherence to follow-up testing recommendations in US veterans screened for lung cancer, 2015-2019. JAMA Netw Open. 2021;4(7):e2116233. doi:10.1001/jamanetworkopen.2021.16233 34236409 PMC8267608

[19] Sakoda LC, Rivera MP, Zhang J, . Patterns and factors associated with adherence to lung cancer screening in diverse practice settings. JAMA Netw Open. 2021;4(4):e218559. doi:10.1001/jamanetworkopen.2021.8559 33929519 PMC8087957

[20] Kunitomo Y, Bade B, Gunderson CG, . Racial differences in adherence to lung cancer screening follow-up: a systematic review and meta-analysis. Chest. 2022;161(1):266-275. doi:10.1016/j.chest.2021.07.2171 34390706

[21] Gudina A, Cupertino A, Kamen CS, . Understanding factors associated with uptake of lung cancer screening among individuals at higher risk. J Clin Oncol. 2021;39(15)(suppl):10559. doi:10.1200/JCO.2021.39.15_suppl.10559 PMC11210810 37464528

[22] Yong PC, Sigel K, Rehmani S, Wisnivesky J, Kale MS. Lung cancer screening uptake in the United States. Chest. 2020;157(1):236-238. doi:10.1016/j.chest.2019.08.2176 31916962 PMC7609956

[23] Leishman NJ, Wiener RS, Fagerlin A, Hayward RA, Lowery J, Caverly TJ. Variation in eligible patients’ agreeing to and receiving lung cancer screening: a cohort study. Am J Prev Med. 2021;60(4):520-528. doi:10.1016/j.amepre.2020.10.014 33342671 PMC10440012

[24] US Census Bureau. Rural-Urban Commuting Area Codes. Published 2019. Accessed July 2, 2020. https://www.ers.usda.gov/data-products/rural-urban-commuting-area-codes/

[25] van Walraven C, Austin PC, Jennings A, Quan H, Forster AJ. A modification of the Elixhauser comorbidity measures into a point system for hospital death using administrative data. Med Care. 2009;47(6):626-633. doi:10.1097/MLR.0b013e31819432e5 19433995

[26] Gould MK, Munoz-Plaza CE, Hahn EE, Lee JS, Parry C, Shen E. Comorbidity profiles and their effect on treatment selection and survival among patients with lung cancer. Ann Am Thorac Soc. 2017;14(10):1571-1580. doi:10.1513/AnnalsATS.201701-030OC 28541748

[27] Wu S, Crespi CM, Wong WK. Comparison of methods for estimating the intraclass correlation coefficient for binary responses in cancer prevention cluster randomized trials. Contemp Clin Trials. 2012;33(5):869-880. doi:10.1016/j.cct.2012.05.004 22627076 PMC3426610

[28] Elwyn G, Frosch D, Thomson R, . Shared decision making: a model for clinical practice. J Gen Intern Med. 2012;27(10):1361-1367. doi:10.1007/s11606-012-2077-6 22618581 PMC3445676

[29] Rivera MP, Tanner NT, Silvestri GA, ; American Thoracic Society Assembly on Thoracic Oncology. Incorporating coexisting chronic illness into decisions about patient selection for lung cancer screening: an official American Thoracic Society research statement. Am J Respir Crit Care Med. 2018;198(2):e3-e13. doi:10.1164/rccm.201805-0986ST 30004250

[30] Kumar V, Cohen JT, van Klaveren D, . Risk-targeted lung cancer screening: a cost-effectiveness analysis. Ann Intern Med. 2018;168(3):161-169. doi:10.7326/M17-1401 29297005 PMC6533918

[31] Lee KT, Harris RP, Schoenborn NL. Individualized approach to cancer screening in older adults. Clin Geriatr Med. 2018;34(1):11-23. doi:10.1016/j.cger.2017.09.002 29129211

[32] Janssen EM, Pollack CE, Boyd C, . How do older adults consider age, life expectancy, quality of life, and physician recommendations when making cancer screening decisions? results from a national survey using a discrete choice experiment. Med Decis Making. 2019;39(6):621-631. doi:10.1177/0272989X19853516 31226903 PMC7080208

[33] Wolf A, Alpert N, Tran BV, Liu B, Flores R, Taioli E. Persistence of racial disparities in early-stage lung cancer treatment. J Thorac Cardiovasc Surg. 2019;157(4):1670-1679.e4. doi:10.1016/j.jtcvs.2018.11.108 30685165 PMC6433143

[34] Lin JJ, Mhango G, Wall MM, . Cultural factors associated with racial disparities in lung cancer care. Ann Am Thorac Soc. 2014;11(4):489-495. doi:10.1513/AnnalsATS.201402-055OC 24701981 PMC5469392

[35] Singh GK, Jemal A. Socioeconomic and racial/ethnic disparities in cancer mortality, incidence, and survival in the United States, 1950-2014: over six decades of changing patterns and widening inequalities. J Environ Public Health. 2017;2017:2819372. doi:10.1155/2017/2819372 28408935 PMC5376950

[36] Alishahi Tabriz A, Neslund-Dudas C, Turner K, Rivera MP, Reuland DS, Elston Lafata J. How health-care organizations implement shared decision-making when it is required for reimbursement: the case of lung cancer screening. Chest. 2021;159(1):413-425. doi:10.1016/j.chest.2020.07.078 32798520 PMC7893305

[37] Goodwin JS, Li S. Clinician and patient characteristics associated with lung cancer screening following a shared decision-making visit. JAMA Netw Open. 2020;3(10):e2021197. doi:10.1001/jamanetworkopen.2020.21197 33074322 PMC7573682

[38] Wiener RS, Koppelman E, Bolton R, . Patient and clinician perspectives on shared decision-making in early adopting lung cancer screening programs: a qualitative study. J Gen Intern Med. 2018;33(7):1035-1042. doi:10.1007/s11606-018-4350-9 29468601 PMC6025674

[39] Draucker CB, Rawl SM, Vode E, Carter-Harris L. Understanding the decision to screen for lung cancer or not: a qualitative analysis. Health Expect. 2019;22(6):1314-1321. doi:10.1111/hex.12975 31560837 PMC6882261

[40] Roth JA, Carter-Harris L, Brandzel S, Buist DSM, Wernli KJ. A qualitative study exploring patient motivations for screening for lung cancer. PLoS One. 2018;13(7):e0196758. doi:10.1371/journal.pone.0196758 29975709 PMC6033377

[41] Slatore CG, Au DH, Press N, Wiener RS, Golden SE, Ganzini L. Decision making among veterans with incidental pulmonary nodules: a qualitative analysis. Respir Med. 2015;109(4):532-539. doi:10.1016/j.rmed.2015.01.007 25660437

[42] Nishi SPE, Lowenstein LM, Mendoza TR, . Shared decision-making for lung cancer screening: how well are we “sharing”? Chest. 2021;160(1):330-340. doi:10.1016/j.chest.2021.01.041 33556362 PMC8295906

[43] Brenner AT, Malo TL, Margolis M, . Evaluating shared decision making for lung cancer screening. JAMA Intern Med. 2018;178(10):1311-1316. doi:10.1001/jamainternmed.2018.3054 30105393 PMC6233759

[44] Kathuria H, Herbst N, Seth B, . Rapid cycle evaluation and adaptation of an inpatient tobacco treatment service at a US safety-net hospital. Implement Res Pract. Published online October 4, 2021. doi:10.1177/26334895211041295 PMC9981890 37089992

[45] Melzer AC, Golden SE, Ono SS, Datta S, Crothers K, Slatore CG. What exactly is shared decision-making? a qualitative study of shared decision-making in lung cancer screening. J Gen Intern Med. 2020;35(2):546-553. doi:10.1007/s11606-019-05516-3 31745852 PMC7018920

[46] National Center for Health Promotion and Disease Prevention. Screening for lung cancer. US Dept of Veterans Affairs. August 25, 2021. https://www.prevention.va.gov/preventing_diseases/screening_for_lung_cancer.asp

[47] Carter-Harris L, Brandzel S, Wernli KJ, Roth JA, Buist DSM. A qualitative study exploring why individuals opt out of lung cancer screening. Fam Pract. 2017;34(2):239-244. doi:10.1093/fampra/cmw146 28122849 PMC6279209

[48] Jonnalagadda S, Bergamo C, Lin JJ, . Beliefs and attitudes about lung cancer screening among smokers. Lung Cancer. 2012;77(3):526-531. doi:10.1016/j.lungcan.2012.05.095 22681870 PMC5055380

[49] Crothers K, Kross EK, Reisch LM, . Patients’ attitudes regarding lung cancer screening and decision aids: a survey and focus group study. Ann Am Thorac Soc. 2016;13(11):1992-2001. doi:10.1513/AnnalsATS.201604-289OC 27652509 PMC5466178

[50] Delmerico J, Hyland A, Celestino P, Reid M, Cummings KM. Patient willingness and barriers to receiving a CT scan for lung cancer screening. Lung Cancer. 2014;84(3):307-309. doi:10.1016/j.lungcan.2014.03.003 24674155 PMC4327769

[51] Wiener RS, Barker AM, Carter-Harris L, . Stakeholder research priorities to promote implementation of shared decision-making for lung cancer screening: an American Thoracic Society and Veterans Affairs Health Services research and development statement. Am J Respir Crit Care Med. 2022;205(6):619-630. doi:10.1164/rccm.202201-0126ST 35289730 PMC12042909

