# Terminology Error

**DOI:** 10.1001/jamanetworkopen.2022.35374

**Published:** 2022-09-16

In the Original Investigation titled “Factors Associated With Declining Lung Cancer Screening After Discussion With a Physician in a Cohort of US Veterans,”^1^ published August 16, 2022, “physician” was changed to “clinician” throughout, and a sentence was added to the Study Setting subsection of the Methods section to indicate that clinicians included physicians, nurse practitioners, and physician assistants. This article has been corrected.^1^
