# Supplementary material for: Factors Associated With Declining Lung Cancer Screening After Discussion With a Physician in a Cohort of US Veterans
Source: JAMA Netw Open. 2022 Aug 16;5(8):e2227126. doi: 10.1001/jamanetworkopen.2022.27126 (PMC9382440; doi:10.1001/jamanetworkopen.2022.27126)
Supplement: Supplement. — eTable 1. International Classification of Diseases, Ninth Revision (ICD-9) and International Statistical Classification of Diseases and Related Health Problems, Tenth Revision (ICD-10) Current Procedural Terminology Codes for Comorbidities eTable 2. Multivariable Analysis of Primary Model Compared With Models Used in Sensitivity Analyses, Including Veterans With Conflicting Eligibility Documentation [file jamanetwopen-e2227126-s001.pdf]

Supplementary Online Content

Núñez ER, Caverly TJ, Zhang S, et al. Factors associated with declining lung cancer screening after discussion with a physician in a cohort of US veterans. *JAMA Netw Open*. 2022;5(8):e2227126. doi:10.1001/jamanetworkopen.2022.27126

eTable 1. *International Classification of Diseases, Ninth Revision (ICD-9)* and *International Statistical Classification of Diseases and Related Health Problems, Tenth Revision (ICD-10)* Current Procedural Terminology Codes for Comorbidities

eTable 2. Multivariable Analysis of Primary Model Compared With Models Used in Sensitivity Analyses, Including Veterans With Conflicting Eligibility Documentation

This supplementary material has been provided by the authors to give readers additional information about their work.

© 2022 Núñez ER et al. *JAMA Network Open*.

eTable 1. *International Classification of Diseases, Ninth Revision (ICD-9)* and *International Statistical Classification of Diseases and Related Health Problems, Tenth Revision (ICD-10)* Current Procedural Terminology Codes for Comorbidities

Comorbidity	ICD-9-CM codes	ICD-10-CM codes	ICD-9 procedure codes	ICD-10 procedure codes	CPT4/HCPCS procedure codes
Elixhauser Comorbodity Index	your code or: http://mchp-appserv.cpe.umanitoba.ca/Upload/SAS/ICD10_EX.sas.txt				
**Other morbidities**					
The next 2 variables are based on a single principal code from inpatient records (VA or Medicare) or primary code from Emergency room data anytime before or on the index date.					
MACE	410, 410.xx, 411.0, 411.1, 411.81, 411.89, 412, 412.xx, 427.5	I20.0, I21, I21.x, I21.xx, I22.x, I24.0, I24.1, I24.8, I24.9, I25.2, I46.9	36.01-36.07, 36.09-36.16, 36.19	0210xxx, 02111xxx, 0212xxx, 0213xxx, 0270xxx, 0271xxx, 0272xxx, 0273xxx	33510-33519, 33521-33523, 33533-33536, 33572, 92973-92975, 92977, 92980-92982, 92984, 92986, 92995, 92996
Stroke, all	430-434, 436, 437.1, 438	I60.9, I61.9, I62.1, I62.00, I62.9, I65.1, I63.22, I66x, I66.xx, I63.30, I64.40, I63.50, I67.8x, I69.9xx			
All other morbidity variables are based on 2+ days with codes (IP or OP, VA or Medicare) in the 731 days (2 years) before and including the index date, except as noted.					
COPD/ Emphysema	491.xx, 492.xx, 496.xx	J40-J44.xx			
Interstitial lung disease	508.1, 515, 516.31, 516.32, 516.34, 518.89, 714.81, 770.7	J70.1, J84.111, J84.112, J84.113, J84.115, J84.9, J98.4, M05.10, P27.0, P27.1, P27.8			
Congestive heart failure	428,428.xx,402.01,402.11,402.91,404.01,404.03, 404.11, 404.13, 404.91, 404.93	I50.x, I50.xx, I11.0, I13.0, I13.2			
HIV	042- 044.xx	B20, B17.1x, B17.8, B17.9, B18.2, B18.8, B18.9			
Dementia, including Alzheimers	290, 290.xx, 331.0-331.82, 331.9, 331.9x, 797	F01.5, F01.5x, F02.8, F02.8x, F03.9, F03.9x, G30.x, G31.x, G31.xx, R41.81			
Other neurodegenerative diseases	340, 340.xx, 332, 332.00	G20, G31.83, G35			
Depression (MDPR+ODPR)	296.2-296.3x, 300.4, 300.4x, 300.9, 300.9x, 301.12, 309.0, 309.0x, 309.1, 309.1x, 309.28, 311, 311.x	F32.x, F32.xx, F33.x, F33.xx, F34.1, F43.21, F43.23, F48.9			
PTSD	309.81	F43.10, F43.11, F43.12			
Schizophrenia	295, 295.xx	F20.x, F20.xx			
Other psychiatric conditions	292.1, 292.1x, 292.81, 292.83, 292.84, 293.0, 293.0x, 293.1, 293.1x, 296-296.1x, 296.4-296.9x, 297-298.xx, 300.0-300.3x, 309.3-309.4x, 312.34	F05, F19.921, F19.94, F19.950, F19.951, F19.96, F22, F23, F28-F31.xx, F32.3, F33.3, F34.0, F34.81, F34.89, F39-F42.xx, F43.24, F43.25, F44.89, F63.81			
Alcohol /Drug Abuse/Use (Substance Use Disorder)	291, 291.xx, 292.0, 292.0x, 292.89, 292.9, 292.9x, 303, 303.xx, 304, 304.xx, 305, 305.0, 305.0x, 305.2-305.9x, 357.5, 425.5, 535.3, 571.0-571.3x, 760.71, 790.3, 977.3, 980.0, 980.9, E860-E860.1, E860.9, E947.3, V11.3, V79.1	F10-F16.xx, F18-F19.xx, F62.1, I42.6, K29.20, K29.21, K70.x, K70.xx, P04.3, Q86.0, R78.0, T50.991A, T51.0X1A, T51.0X2A, T51.0X3A,T51.0X4A, T51.91XA, T51.92XA, T51.93XA, T51.94XA, NOD.X, Z65.8, Z13.89			

© 2022 Núñez ER et al. *JAMA Network Open*.

eTable 2. Multivariable Analysis of Primary Model Compared With Models Used in Sensitivity Analyses, Including Veterans With Conflicting Eligibility Documentation

	Primary Model (N=43.450)			Sensitivity model including Veterans with conflicting eligibility (N=50,345)		
	OR	95% CI		OR	95% CI	
Age (65-69 as reference)						
55-59	**0.69**	0.64	0.74	**0.69**	0.64	0.73
60-64	**0.80**	0.75	0.85	**0.82**	0.77	0.87
70-74	**1.27**	1.19	1.37	**1.24**	1.17	1.33
75-80	**1.93**	1.73	2.17	**1.82**	1.64	2.02
Female	1.08	0.95	1.24	1.08	0.95	1.22
Race (white as reference)						
Black	**0.80**	0.73	0.87	**0.80**	0.74	0.86
Hispanic	**0.62**	0.49	0.78	**0.61**	0.50	0.75
Other	0.98	0.80	1.20	0.91	0.75	1.10
Married	1.05	0.99	1.10	1.04	0.99	1.09
Zip code-level income*	0.95	0.87	1.03	0.96	0.89	1.03
Live in rural zip code	1.01	0.95	1.08	1.01	0.95	1.07
Distance to VA Facility*	1.06	1.03	1.08	1.06	1.03	1.08
VA Benefits (Limited with copayments as reference)						
Highly disabled	0.94	0.87	1.02	0.94	0.87	1.01
Low or moderately disabled	**0.89**	0.82	0.96	**0.89**	0.83	0.96
Poverty with no copayments	**0.92**	0.85	0.99	**0.92**	0.86	0.99
Comorbid conditions						
Currently smoking	**0.90**	0.85	0.96	**0.90**	0.85	0.95
History of major adverse cardiac event	0.95	0.88	1.04	0.98	0.90	1.05
Congestive heart failure	**1.25**	1.12	1.39	**1.23**	1.11	1.36
Stroke	**1.14**	1.01	1.28	**1.13**	1.01	1.26
Chronic obstructive pulmonary disease	1.00	0.94	1.06	0.99	0.94	1.05
Interstitial lung disease	0.98	1.44	1.21	**1.02**	1.43	1.27
Dementia	1.03	0.88	1.21	1.05	0.91	1.21
Depression	**0.86**	0.80	0.92	**0.86**	0.81	0.91
Post-traumatic stress disorder	0.95	0.88	1.03	0.93	0.87	1.01
Substance use disorder	0.95	0.89	1.02	0.95	0.89	1.01
Schizophrenia	**1.87**	1.60	2.19	**1.82**	1.58	2.09
Elixhauser Comorbidity Index score	**1.04**	1.03	1.05	**1.04**	1.03	1.05
Human Immunodeficiency Virus	0.93	0.80	1.08	0.93	0.81	1.07
**Healthcare Utilization in the year before index date***						
Outpatient visits	**0.70**	0.67	0.72	**0.70**	0.68	0.73
Emergency department visits	**0.86**	0.80	0.92	**0.87**	0.82	0.92
Inpatient days	1.04	0.99	1.10	1.03	0.99	1.08
Long-term care facility days	**1.07**	1.19	1.13	**1.08**	1.19	1.16
**Facility-level characteristics**						
US Census region (Northeast as reference)						
Midwest	1.56	0.26	9.60	1.38	0.25	7.56
South	1.17	0.24	5.66	1.06	0.24	4.59
West	0.93	0.18	4.88	0.79	0.17	3.73
Lung cancer screening volume (high volume as reference)						
Low	3.74	0.72	19.41	3.06	0.65	14.36
Medium	2.00	0.42	9.64	1.92	0.44	8.35

*** Bolded Odds ratio (OR) are statistically significant at alpha = 0.05**

© 2022 Núñez ER et al. *JAMA Network Open*.
